# Geometry impact on fundamental properties of theophylline-containing SLS printed pharmaceutical tablets

**DOI:** 10.3389/fddev.2024.1358336

**Published:** 2024-03-06

**Authors:** Valerie R. Levine, Christos S. Katsiotis, Maria Strømme, Julian Quodbach, Jonas Lindh

**Affiliations:** ^1^ Division of Nanotechnology and Functional Materials, Department of Material Science and Engineering, Uppsala University, Uppsala, Sweden; ^2^ Department of Pharmaceutics, Utrecht Institute for Pharmaceutical Sciences, Utrecht University, Utrecht, Netherlands

**Keywords:** selective laser sintering (SLS), additive manufacturing (AM), surface area-to-volume ratio (SA/V), oral dosage forms, geometries

## Abstract

Selective Laser Sintering (SLS) has the potential to offer a more accurate alternative to current-practice manipulation of oral dosage forms for pediatric, geriatric, and dysphagia-suffering patient groups. In order to create the best possible dosage forms for these patient groups, an in-depth look into how a dosage forms geometry impacts the overall properties is essential. In this study, the impact of geometry on SLS manufactured oral dosage forms on the tablet’s microstructure, actual-to-theoretical volume, mass deviation, disintegration, and dissolution was investigated. Three different shapes; cylinder, hollow cylinder, and conical frustum with similar surface area (SA), as well as three cylinders with different diameters, were investigated. The results indicate that the geometry has an impact on the mass uniformity, resultant volume, disintegration, and dissolution properties of the tablets. The mass uniformity analysis of the tablets provided the most variation between tablets of different sizes, with more uniformity for tablets with similar SA-to-volume ratio (SA/V). When examining the actual-to-theoretical volume of the tablets, a greater variance between the actual and theoretical volumes for shapes with higher overall SA was observed. The values found are approximately 1.05 for the three differently sized cylinders, 1.23 for the conical frustum, and 1.44 for the hollow cylinder, following this trend. Disintegration data supported a link between SA/V and average disintegration time, observed with the tablet of the highest SA/V disintegrating in 12 s and the tablet with the lowest SA/V disintegrating in 58 s. Dissolution results also indicated a strong dependence on SA/V. Hence, when novel ways to produce oral dosage form tablets become available by additive manufacturing, such as SLS, both geometry and SA/V must be taken into consideration in the tablet design process to ensure appropriate release kinetics and dosing standards.

## 1 Introduction

Additive manufacturing (AM) is gaining ground for production of oral dosage forms, particularly for specific patient sets, including pediatric, geriatric, and dysphagia-suffering patient groups. These patient groups struggle with conventional oral dosage forms, particularly the pediatric population, as conventional oral dosage forms are not intended for this age group (Waller, 2007; Zahn et al., 2020). Due to the lack of appropriate medication for this patient subset, manipulation of oral dosage forms is required (Richey et al., 2013; van der Vossen et al., 2019). This manipulation is currently often done by hand, with a kitchen knife, tablet splitter, crushing or milling, or by dispersing the tablets in water and administering a portion of the liquid suspension (Verrue et al., 2011; Logrippo et al., 2017; Zahn et al., 2020). The accuracy of these methods cannot be assured (Kaushal et al., 2001; Walsh et al., 2018), indicating that patient groups reliant on the manipulation of oral dosage forms may not receive a consistently accurate dosage of medication. An inaccurate dosage poses a potential risk of harm to the patient.

AM has the potential to manufacture oral dosage forms for these patient groups. Selective laser sintering (SLS) technology has shown great promise in this field in recent years. This technology, patented in 1989 (Carl, 1989), has been used for many other applications, such as producing prototypes and rapid tooling (Kumar, 2003). SLS is a powder bed fusion (PBF) process, by which a powder is sintered together via a laser, either diode- or CO_2_-based, in a layer-by-layer fashion. After each layer is sintered together, a recoater applies a new powder layer of a select thickness on top of the previous layer, until the part is complete. This process occurs in a build chamber where a selected temperature is applied, keeping an elevated temperature throughout the build. These parts are fully immersed in the powder during the print, so complex shapes and geometries are possible to produce, as no additional support structures are needed (Carl, 1989; Gibson et al., 2015; Kutz, 2017). Also, SLS requires no complex post-processing steps and provides the possibility to reuse unsintered powder material (Gokuldoss et al., 2017). Additionally, this technique provides tablets that look similar to traditionally-pressed tablets, as it is a powder-based technique, which leads to a higher acceptance of the resultant oral dosage forms than with other AM methods, such as fused deposition modelling (FDM) (Januskaite et al., 2020). However, no SLS printer currently on the market is specifically designed for pharmaceutical applications. Nevertheless, there has been a plethora of publications on SLS for oral dosage forms, with the concept of employing SLS for pharmaceutical applications emerging around the turn of the new millenium (Wu et al., 1996; Leong et al., 2001). Publications on the topic have grown significantly since 2017, when SLS was first reported to produce oral drug loaded products (Fina et al., 2017). These publications explore several areas, including complex porous structures for tailored drug release (Fina et al., 2018a) and the impact of different print parameters (Barakh Ali et al., 2019; Mohamed et al., 2020; Tikhomirov et al., 2023a).

Geometry is an especially important aspect to consider when producing oral dosage forms, and this is no different for SLS printed dosage forms. Ideal dimensional accuracy has been investigated for different AM techniques, with SLS often described as having higher dimensional accuracy than other methods (Kluska et al., 2018; Kozak and Zakrzewski, 2018; Msallem et al., 2020). Nevertheless, with this technique still being relatively new, it is important to show how printing data differs from that of theoretical or expected data. There are many aspects to consider that are not present in traditional compounding (i.e., printer settings such as laser scan speed and layer height for SLS printing), which could have an impact on the resultant tablets in terms of the mass and volume of resultant prints (Tikhomirov et al., 2023b). Pharmacopoeia has quality control standards for conventional dosage forms, but without this regulation over AM oral dosage forms, it is especially important to have uniformity in mass and volume and understand the deviations that may arise between expected or theoretical data and resultant data. Divergences from expectations can cause dosing errors or differences in dissolution behavior, which could potentially cause harm to vulnerable patient groups. If these deviations could be mitigated, it could aid in the acceptance of this technique for the manufacturing of oral dosage forms. Therefore, it is paramount to closely investigate the full impact that geometry has on SLS printed oral dosage forms.

The effect of the shape and surface-area-to-volume ratio (SA/V) on characteristics of tablets printed via SLS is not yet investigated to the extent necessary to show the legitimacy of this method as a viable option for the manufacturing of oral dosage forms. While publications of SLS for oral dosage forms have dealt with variations in shape, they have focused on topics such as gyroid lattice structures for tailored drug performance (Fina et al., 2018a) or novel shapes in relatively small quantities (Awad et al., 2020). The ability to produce consistent tablets of a desired shape and understand the consequences of the choice of geometry has not been covered but is vital for the acceptance of this technique for pharmaceutical applications. By examining different shapes designed to have roughly the same surface area (SA), as well as analyzing the impact of the SA/V on tablets of the same shape with variability in diameter, any impacts can be applied to future tablet design. Such impacts could be related to the tablet uniformity and expected dimensions, internal structure of the tablets, microstructure, disintegration, and dissolution properties. Shapes and sizes designed to be within a realistic, swallowable range were chosen, as they hold the most relevance for the adoption of this method for pharmaceutical application. The results of this study will show whether the geometry of a tablet impacts its properties, as well as show signs of print quality consistency for this method. These are important steps and pieces of knowledge for a new field such as SLS for oral dosage forms.

## 2 Materials and methods

### 2.1 Materials

Theophylline anhydrous powder, produced by Sigma-Aldrich Chemie GmbH, was used as the active pharmaceutical ingredient (API) in this study (Steinheim, Germany). Activated carbon, produced by Sigma-Aldrich Chemie GmbH, was used as colorant (Steinheim, Germany), silicon dioxide colloidal (EMPROVE^®^ ESSENTIAL, Ph. Eur., JP, NF, E 551), copolymer of 1-vinyl-2-pyrrolidone and vinyl acetate (copovidone, Kollidon ^®^ VA 64, PVP-VA) was kindly provided by BASF (Ludwigshafen, Germany).

### 2.2 Formulation Preparation

The formulation of the powder was prepared according to Table 1. The excipient was weighed, manually mixed, and sieved using a 315 µm stainless-steel test sieve (VWR International AB, Stockholm, Sweden). The sieved formulation was then mixed further with a Turbula shaker (Turbula T2F shaker, Glen Mills, Inc., Clifton, NJ, US) for 20 min to ensure a resultant homogeneous powder. Pigment was added to the powder formulation to enhance the laser energy absorption, while colloidal silica was added to improve the powder flowability during the layer application process of the print. After mixing, the powder was heat treated overnight at 70°C in an oven (Incucell^®^, BMT Medical Technology s.r.o., Brno, Czech Republic). This was done to remove excess moisture, with a temperature well below the Tg for copovidone but high enough to remove as much moisture as possible. The formulation was prepared in a batch of 1,252.5 g, or approximately 1,500 mL. The formulation was made up by weight percent of 88.5% polymer, 10% API, 1% pigment, and 0.5% silicon dioxide.

**TABLE 1:** Composition of prepared powder formulation.

Copovidone (g)	Pigment (g)	Silicon dioxide (g)	Theophylline (g)
1,108.5	12.5	6.3	125.2

### 2.3 Shape and size selection of tablets

For the first part of the study, a 10 mm diameter x 4 mm height cylindrical tablet was chosen as the basis for the study. All shapes were designed using Fusion 360 (Student Edition, Autodesk, USA). This shape and size of tablet was chosen due to the realistic nature, with it being a size and shape that could easily be ingested. This study intends to compare the effect of the shape of the tablet, as well as examine the impact of SA/V. The shapes chosen for the different geometries all had approximately the same SA. Dimensions were chosen according to printer resolution and materials used. Aside from the base cylinder with 10 mm diameter x 4 mm height, a hollow cylinder with an outer diameter of 11 mm, an inner diameter of 6 mm, and a height of 3 mm was chosen. Additionally, a conical frustum was chosen with a top diameter of 8 mm, a bottom diameter of 11 mm, and a height of 4 mm. For the shapes, dimension details can be seen in Table 2.

**TABLE 2:** Surface area (SA), volume, surface-area-to-volume ratio (SA/V), and formulas for the various shapes investigated.

Shape	SA (mm^2^)	Volume (mm^3^)	SA/V	Formulas for SA (A_S_) and volume (V)
Cylinder	282.7	314.2	0.9	$A_{s}=2\pi rh+2\pi r^{2}$
				$V=\pi r^{2}h$
Hollow Cylinder	293.7	200.3	1.5	$A_{s}=2\pi \left({r_{1}}^{2}-{r_{2}}^{2}\right)+2\pi \left(r_{1}+r_{2}\right)h$
				$V=\pi \left({r_{1}}^{2}-{r_{2}}^{2}\right)h$
Conical Frustum	272.8	285.9	0.95	$A_{s}=\pi \left({r_{1}}^{2}+{r_{2}}^{2}+\left(r_{1}+r_{2}\right)*{\sqrt{r_{1}-r_{2}}}^{2}+h^{2}\right)$
				$V=\frac{1}{3}\pi h\left({r_{1}}^{2}+{r_{2}}^{2}+\left(r_{1}*r_{2}\right)\right)$

For the second part of the study, cylinders of varying diameter, 6 mm, 10 mm, and 14 mm with a height of 4 mm were designed. This allowed for tablets with a noticeable difference in SA/V (Table 3).

**TABLE 3:** Surface area, volume, and surface-area-to-volume ratio for the selected cylinder sizes.

Cylinder diameter	SA (mm^2^)	Volume (mm^3^)	SA/V (mm^−1^)
Cylinder (d = 6 mm)	131.9	113.1	1.17
Cylinder (d = 10 mm)	282.7	314.2	0.9
Cylinder (d = 14 mm)	483.8	616.8	0.79

### 2.4 Selective laser sintering of dosage forms

**FIGURE 1:**
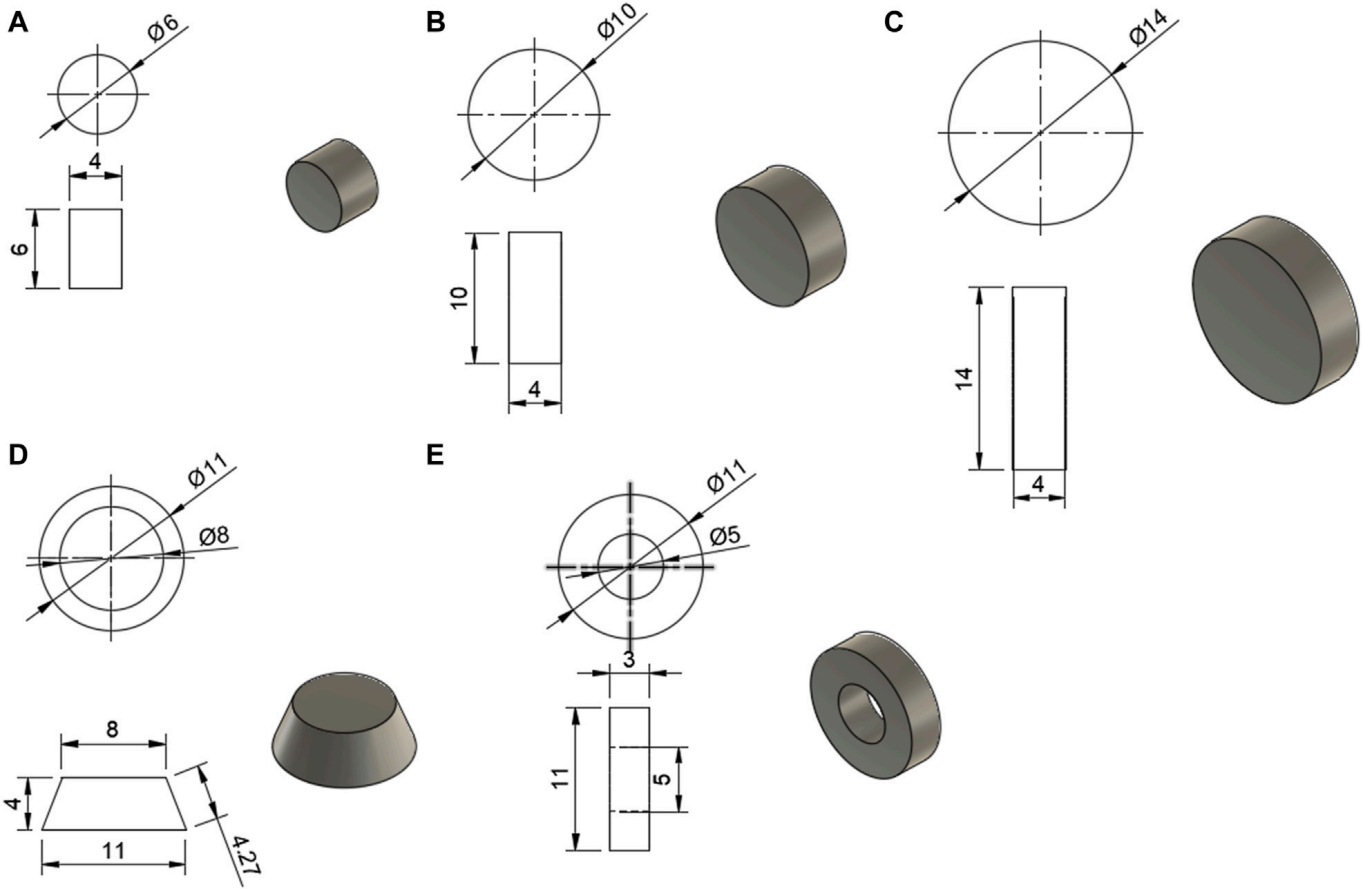
Models for the tablets, with all dimensions in mm; **(A)** 6 mm cylinder, **(B)** 10 mm cylinder, **(C)** 14 mm cylinder, **(D)** conical frustum, **(E)** hollow cylinder.

The models for the tablets were designed in Fusion 360 (Student Edition, Autodesk, USA) and are shown in Figure 1. These models were then uploaded as stereolithography (STL) files to Sintratec Central 1.2.7 (Sintratec AG, Brugg, Switzerland).

The printer used for this study was a Sintratec Kit printer (Sintratec AG, Brugg, Switzerland). For the 3D printing process, the prepared powder formulation was loaded into the powder reservoir platform, with chamber dimensions of 100 × 100 × 100 mm. A thin layer of the powder formulation was spread over the build platform and the chamber and powder beds were gradually heated to 100 °C and 70°C, respectively. The sintering process was carried out with a 2.3 W diode laser (λ = 455 nm) in accordance with the template models given in the STL-file in a layer-by-layer fashion. All tablets were printed with a 50 μm perimeter offset, 50 μm hatching space, and 150 μm hatching offset, as well as three perimeter paths. The total number of tablets deviated per batch, as each shape differed in dimensions. A layer height of 150 µm and a laser speed of 300 mm/s were chosen for the batches based on pretrials with the powder formulation. After completed printing and the printer cooling cycle, the batches were collected from the build platform via sieving. To remove as much excess powder as possible, the tablets were further dedusted using pressurized air. They were then stored in sealed containers until analysis and characterization was performed. For each of the five selected shapes and sizes of tablets, three batches were printed.

### 2.5 Solid-state analysis

Differential scanning calorimetry (DSC) thermograms were obtained using a Mettler Toledo DSC 3+ (Schwerzenbach, Switzerland) with a heating and cooling rate of 10°C min^−1^ and nitrogen as a purge gas. Heating-cooling measurements were carried out from −40°C to 300°C and from 300°C to 10°C in the first cycle, and from 10°C to 300°C in the second cycle.

Powder X-ray diffraction (PXRD) diffractograms of the heat-treated powder formulation, as well as the printed dosage forms, were collected on the D8 Advance TwinTwin X-ray diffractometer (Bruker AXS GmbH, Bremen, Germany) using Cu-Kα1,2 (λ = 1.5418 Å) radiation. The instrument was operated at 40 mA and 40 kV with a step-size of 0.02° and a data collection time of 1 h.

### 2.6 Physical characteristics

The dimensions and weights of the printed tablets were examined using a digital caliper (digital sliding caliper 150 mm stainless steel, Distrelec Group AG, Nänikon, Switzerland) and an analytical balance (Mettler Toledo XS 64 Analytical Balance, Schwerzenbach, Switzerland). The number of tablets printed differed for each shape, as the number of tablets printed per batch changed to optimize the print bed space. The total number of tablets measured and weighed for each shape is shown in Table 4.

**TABLE 4:** Total number of tablets measured with caliper and weighed with an analytical balance over the 3 batches printed for each shape.

	Cylinder (d = 6 mm)	Cylinder (d = 10 mm)	Cylinder (d = 14 mm)	Hollow cylinder	Conical frustum
Total number of tablets measured with caliper	n = 30	n = 30	n = 30	n = 30	n = 30
Total number of tablets weighed with analytical balance	n = 126	n = 90	n = 36	n = 72	n = 63

Micro-CT measurements were performed on a CT-Alpha (Procon X-Ray, Sarstedt, Germany) to analyze the tablet composition for each different tablet geometry and size. A voxel resolution of 10 µm was used (12 µm for 14 mm cylinder), with an x-ray tube current of 30 mA and a voltage of 80 kV. Image stacks of 1,600 projections were used and each projected image had an exposure time of 500 ms. Particle size measurement of the polymer powder was also performed using a Mastersizer 3,000 (Malvern Panalytical Ltd., Malvern, United Kingdom) with ten measurements for each sample with a 10 s measurement duration. The result is presented as volume-based.

### 2.7 Disintegration

Disintegration testing was performed on a PTZ Auto EZ fully automated tablet disintegration testing apparatus from Pharmatest (Hainberg, Germany). This testing was done in accordance with Phr. Eur. 2.9.1 Disintegration of Tablets and Capsules Test A. 450 mL of 37 °C milliQ water was used as the disintegration medium.

### 2.8 Drug content

Theophylline was dissolved in phosphate buffer, pH 6.8, at room temperature and its spectrum (200–600 nm) was recorded on a UV-VIS spectrophotometer (1800, Shimadzu Corporation, Kyoto, Japan). A calibration curve of the drug in phosphate buffer was prepared in the range of 0.5–15 mg/L and measured at 271 nm (*R* ^2^ > 0.99).

### 2.9 *In vitro* drug release

Drug release studies were performed on the five different samples, i.e., cylinder (d = 6 mm, d = 10 mm, d = 14 mm), hollow cylinder, and conical frustum. The tests were executed according to the USP II method with a Sotax AT7 (Sotax AG, Aesch, Switzerland). Each vessel was filled with 900 mL of phosphate buffer, pH 6.8, and was kept at 37°C and at 100 rpm stirring. At predetermined time intervals 3 mL sample aliquots were removed and filtered with 0.45 μm PTFE filters. Fresh buffer of equal volume was used to replenish the release medium. All experiments were performed in triplicate. A control sample was used containing all additives, in corresponding quantities, except the drug. The percentage and mass of the drug released was calculated via measuring the sample aliquots with UV-VIS spectroscopy. The drug release profiles were investigated for fit in two kinetic profiles; first-order and Higuchi, using DDsolver (Zhang et al., 2010).

## 3 Results and discussion

### 3.1 Pure polymer analysis

The polymer particle size distribution for the copovidone used in this study, seen in Table 5, is an important factor for the printability of a material. In this case, the results align with findings from previous publications (Chaudhary et al., 2018). Additionally, copovidone was used in this study due to the frequency of usage in other SLS publications, highlighting its suitability for SLS (Allahham et al., 2020; Awad et al., 2020; Mohamed et al., 2020; Davis et al., 2021). It is important to know the particle size going into printing, as if the particles are too large compared to the layer height, the printing will not succeed. A layer height of 150 µm was chosen, as it is at the x90 of the size distribution, and it is the maximum layer height that the printer will allow. This layer height and material choice were verified during pretrial printing to have proper recoating of new surface layers.

**TABLE 5:** Particle size distribution.

	x10 (µm)	x50 (µm)	x90 (µm)
copovidone	27.3 ± 0.7	79.5 ± 1.3	150 ± 10.1

### 3.2 Visual properties of tablets

#### 3.2.1 Visual analysis

**FIGURE 2:**
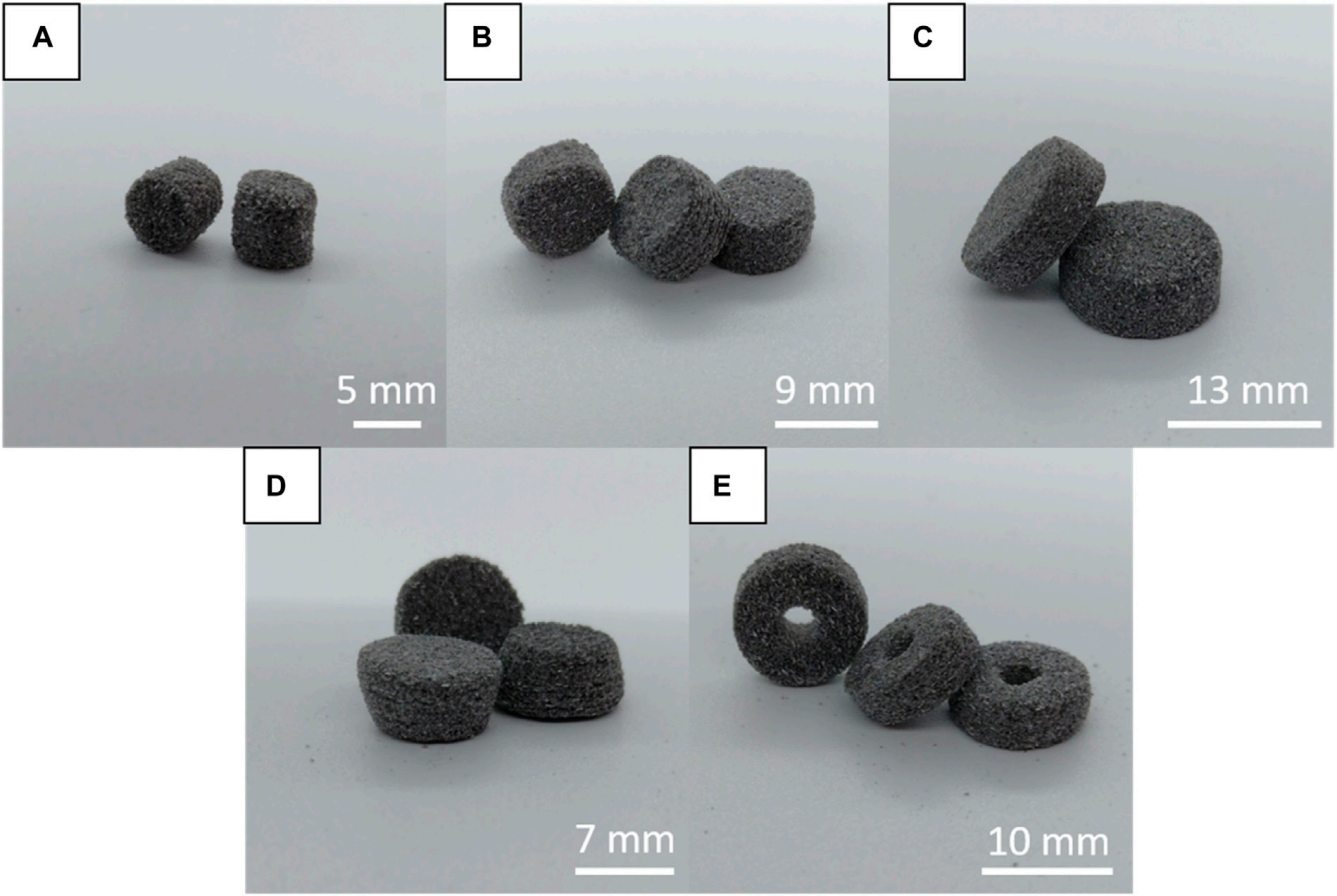
Printed tablets; **(A)** cylinders (d = 6 mm), **(B)** cylinders (d = 10 mm), **(C)** cylinders (d = 14 mm), **(D)** conical frustums, **(E)** hollow cylinders.

**FIGURE 3:**
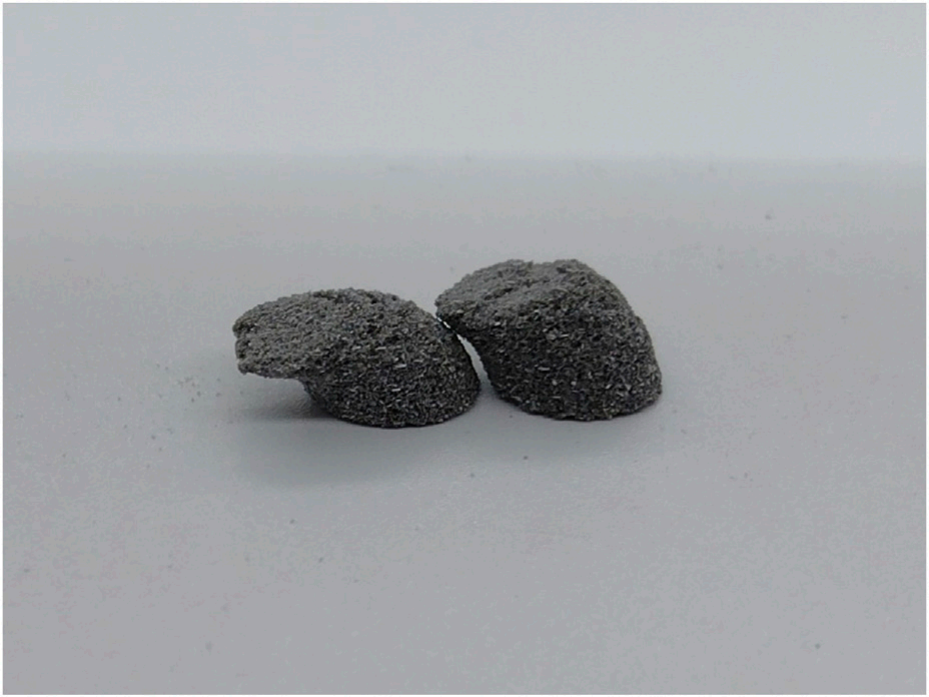
Example of tablets that exhibit shifting of the layers during recoating and over-sintering.

The tablets all exhibited the same general aesthetics, as is seen in Figure 2. The tablets have distinct layers visible in some cases, particularly the 10 mm diameter cylinder tablet in the center of the image in part B of Figure 2. Visually, the level of sintering and color give no indication of differences in terms of the print quality. While a qualitative analysis of the images does not give a deep level of information regarding the tablet characteristics, it can show a clear, initial indication that sintering had gone amiss, the print temperature had been wrong for the formulation, or the laser scan speed had been too high or too low. The laser scan speed must be higher than for other pigments, such as red pigments like Candurin^®^ NXT Ruby Red (Hamed et al., 2021), as it was observed to over-sinter with too low laser scan speeds. Black pigment absorbs more energy compared to lighter colored pigments. This leads to more efficient energy transfer and, therefore, more energy at the same scan speed than for a lighter color. This was also the case for the formulation used in this study, which required adjustments of both temperature and laser scan speed on a trial-and-error basis after initial testing with conditions previously found advantageous in literature (Tikhomirov et al., 2023b). The temperature needed to be lowered and the laser scan speed needed to be increased from what was previously reported using red pigment on the Sintratec Kit printer (Tikhomirov et al., 2023b). Incorrect print conditions can cause the layers of the tablets to warp or drag and shift. Resulting tablets from a trial with over-sintering and layer shifting can be seen in Figure 3. Dragging occurred during the recoating process between layers.

#### 3.2.2 Tablet imaging with Micro-CT

**FIGURE 4:**
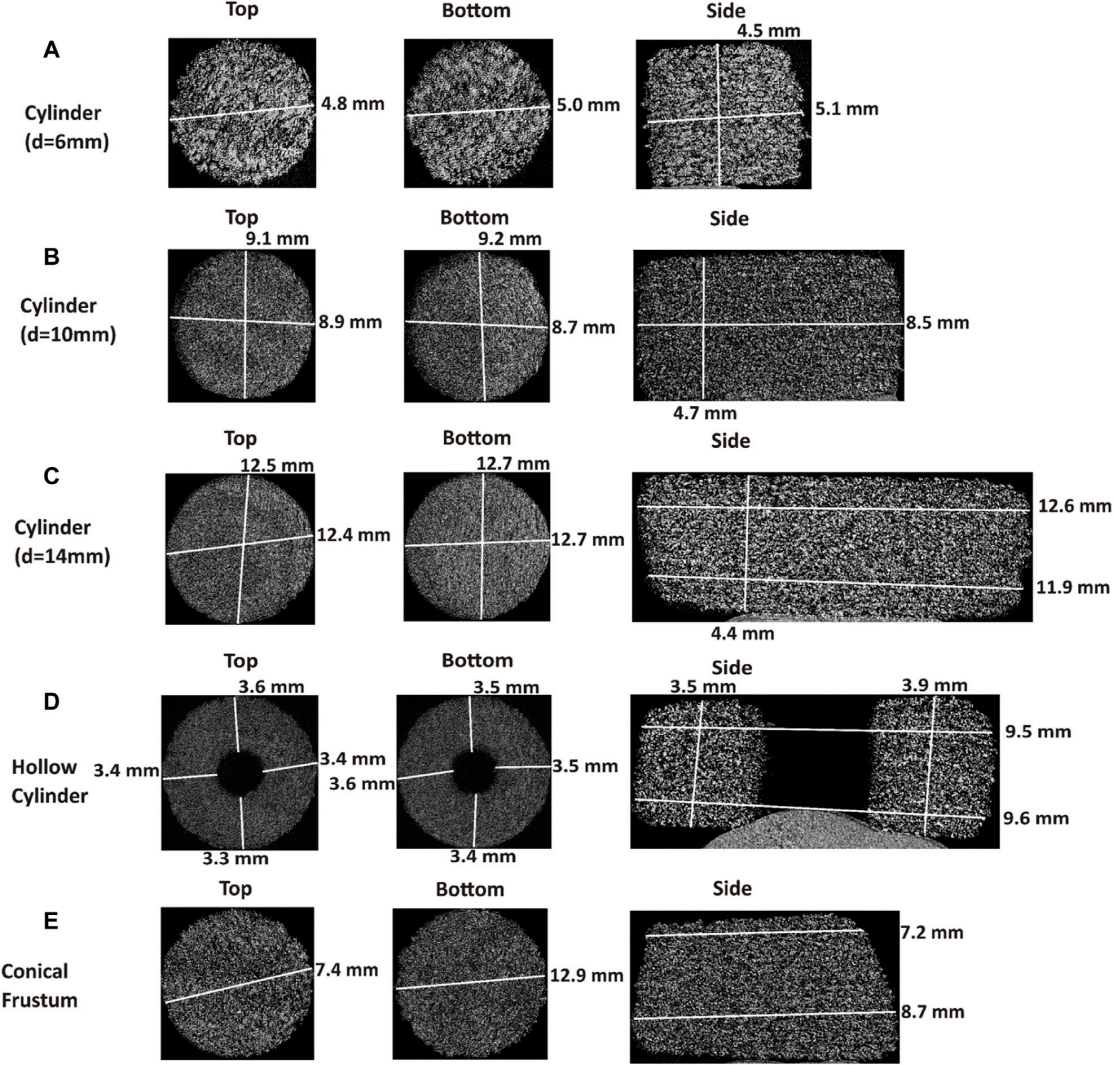
Top, bottom, and side micro-CT images of **(A)** cylinder (d = 6 mm), **(B)** cylinder (d = 10 mm), **(C)** cylinder (d = 14 mm), **(D)** hollow cylinder, **(E)** conical frustum.

Analyzing the micro-CT images, seen in Figure 4, the internal structure for the different shapes and cylinder sizes can be observed. The 6 mm diameter cylinder showed a fairly consistent size from top to bottom and the print resolution appears to be rather good. However, distinct layers are visible, which could be indicative of a less-than-perfect sintering between the layers. The 10 and 14 mm diameter cylinders also showed some distinct layers, which is similarly observable in the images in Figure 2. The lack of discernible difference between the bulk of the 10 and 14 mm diameter tablets is interesting, as they are visually distinct from each other in Figure 2. The 10 mm diameter cylinder has layers more clearly visible than the 14 mm diameter cylinder, yet, the bulk of these tablets looks similar. The resolution of the sides of the tablets, as is the case with a majority of the tablets analyzed, is not perfectly sharp. This is likely due to the particle size of the powder and the laser spot size of the printer, as both of these factors have an impact on the resultant resolution. The particle size of the copovidone (x50 of around 80 µm) is slightly larger than that of commercial PA12 (Obst et al., 2018; Xu et al., 2019) (x50 of around 60 µm), and will therefore yield a courser final resolution. Additionally, there are some adjacent particles attached to the surface of the tablets that are intended to be sintered. With SLS, the printed object is surrounded by unsintered powder, where some of that powder has the possibility to stick to the textured outer surface of the tablets even after dedusting. This is, therefore, a result of the method. The hollow cylinder has very good resolution from a top and bottom cross section. From a side cross section, this is not as consistent. The thickness deviates by 400 µm. This could potentially be due to the amount of perimeter sintering occurring for this shape, which has the highest SA/V. This larger proportion of sintering could have led to some inconsistent sintering from the perimeter to the interior of the tablet. The more sintering that occurs, the more likely the material is to form a denser structure. The conical frustum exhibits some visible layers, with the bulk of the tablet appearing much like that of the 10 and 14 mm diameter tablets. This could be due to this tablet being similar to these cylinders, both in regard to size and shape. The conical frustum is more similar to that of a cylinder in terms of SA/V, and the thickness of the tablet was intended to be the same as for the cylinders. Therefore, the micro-CT images looking more similar is not an unexpected outcome.

### 3.3 Solid-state characterization

**FIGURE 5:**
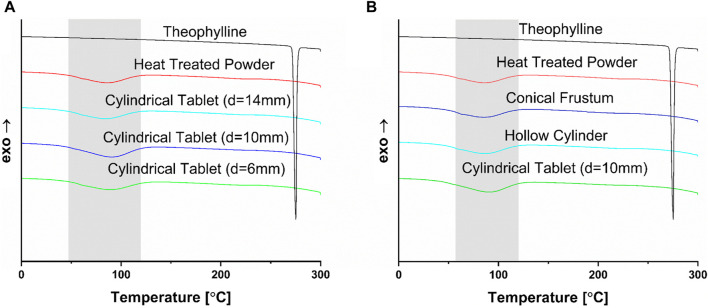
**(A)** DSC data of selected sizes of cylinder (d = 6 mm, d = 10 mm, d = 14 mm) compared to the heat-treated powder mixture and pure theophylline; **(B)** DSC data of selected shapes (cylinder, hollow cylinder, conical frustum) compared to the heat-treated powder mixture and pure theophylline.

**FIGURE 6:**
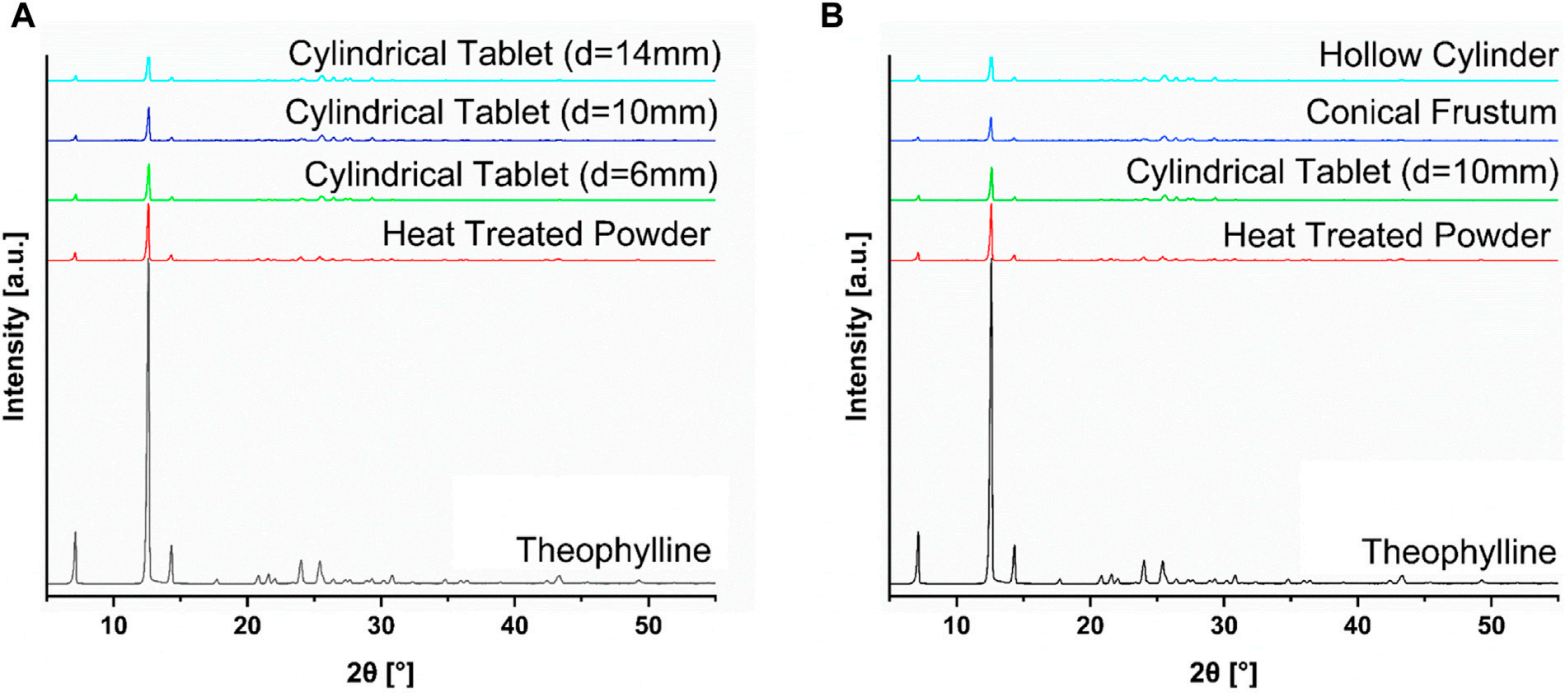
**(A)** XRD data of selected sizes of cylinder (d = 6 mm, d = 10 mm, d = 14 mm) compared to the heat-treated powder mixture and pure theophylline; **(B)** XRD data of selected shapes (cylinder, hollow cylinder, conical frustum) compared to the heat-treated powder mixture and pure theophylline.

The DSC results, shown in Figure 5, have no indication of crystallinity, with no signals for the API in any of the tablets. It is logical that these peaks would not show up since the powder was heat-treated at 70 °C overnight, leading to the disappearance of the theophylline peak prior to any printing, as the peak is already absent in the heat-treated powder. From the DSC results, the evaporation event of water, shown in grey in Figure 5, is visible from around 60°C–120 °C. It is important to note the occurrence of this thermal event, despite the fact that it does not concern the API. However, the XRD results in Figure 6 show a different perspective for the solid-state characteristics of the tablets.

According to the XRD results, all tablets show signs of crystallinity, with distinct peaks relating to the API. Therefore, the tablets manufactured contained crystalline API. For ideal drug delivery, it would be best to have tablets containing amorphous API. For theophylline, this is not a concern, as this API is fairly water soluble, but could be important for water-insoluble APIs. Additionally, this study focuses on the reproducibility of the tablets in different geometries as well as the differences between tablets when changing the shape or SA/V. For these research questions, the properties of the solid-state were less decisive as long as results were comparable for all printed geometries. Since all of the tablets exhibit the same distinct peaks relating to the API, there is no major difference in these tablets with respect to the solid-state properties.

**FIGURE 7:**
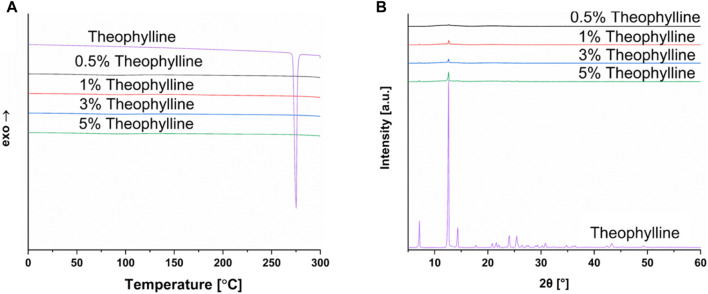
**(A)** DSC of theophylline in different amounts (0.5%–5%) in copovidone powder compared to pure theophylline, **(B)** XRD of theophylline in different amounts (0.5%–5%) in copovidone powder compared to pure theophylline.

The differences found between the DSC and XRD analysis indicates the importance of utilizing two independent methods to investigate the solid-state properties of materials. The authors could not find literature data for theophylline indicating a similar discrepancy between DSC and XRD results. Therefore, small quantities of theophylline were mixed with copovidone and characterized to examine the differences between the DSC and XRD methodology, and at what level theophylline peaks still appear in XRD thermograms. Results are shown in Figure 7. This analysis indicated that even at very low concentrations, such as 0.5% theophylline content, peaks indicative of crystalline theophylline are still present. This is not the case for DSC analysis, emphasizing the importance of utilizing both methods for solid-state characterization. The finding of DSC and XRD having discrepancies is observed in other publications, including in the field of SLS for oral dosage forms (Tikhomirov et al., 2023b).

### 3.4 Mass analysis of tablets

The number of tablets produced in this study for each shape investigated are shown in Table 6. It is important to note the damaged tablets, either via delamination, severe shifting between the layers, or breakage while handling, as it shows the reproducibility of the printing of each geometry in this study. Inconsistencies could have arisen from the variation in temperature that occurs in the printing chamber during the printing process. The temperature is not completely consistent during printing in one spot, let alone across the entire print bed surface. Due to uneven print bed temperature, the print could have been compromised (Tikhomirov et al., 2023c).

**TABLE 6:** Number of damaged tablets, total tablets, and percentage of tablets damaged for each shape investigated.

Shape	Number of tablets damaged	Total tablets printed (all 3 batches)	Print failures (%)
Cylinder (d = 6 mm)	10	126	7.94
Cylinder (d = 10 mm)	1	90	1.11
Cylinder (d = 14 mm)	0	36	0.00
Hollow Cylinder	1	72	1.38
Conical Frustum	1	63	1.59

The 6 mm diameter cylinder had a much higher percentage of tablets damaged per the total printed than any other shape. Comparing the 6 mm diameter tablet to the other tablets, the print failure was much higher. All other shapes had print failure percentages below 2%. The exact reason for this is not known, though it could be related to the smaller size of the tablet and the lower densification of the tablet during printing. A larger layer has more heat energy absorbed, and has the potential for higher part densification, whereas the 6 mm diameter tablet could have undergone less densification and therefore more damaged tablets. Additionally, it is possible that more layer dragging occurred when the recoater applied new layers to the print bed of the printer due to the small size of these tablets.

Table 7 shows the average mass, coefficient of variation, percent outliers of intact tablets, and the percentage outliers including damaged tablets. For tablets with an average mass between 80 and 250 mg, the allowable percent deviation in the mass per tablet, according to Phr. Eur. 2.9.5 uniformity of mass of single-dose preparations, is 7.5%. This applies to all tablets in this study, except for the cylinders with diameters of 6 and 14 mm. The tablets with a diameter of 6 mm are below 80 mg, and can therefore have a 10% deviation. The 14 mm diameter tablets, which are above 250 mg, may have a deviation of no more than 5%. No more than two of these tablets, per batch, are allowed to deviate in excess of these percentages. Pharmacopeia requirements would dictate weighing 20 units at random. However, each tablet produced and undamaged was weighed. Since this printing method, as well as any other AM printing method, does not yet have specific guidelines, it was decided that all of the intact tablets printed for each size and shape would be investigated. This would allow for transparency on the quality of the printing and aid with future work and regulatory quandaries in this field.

**TABLE 7:** Mass data for different shapes (n ≥ 12, average ±sd).

Shape	Average Mass (mg) ± sd (%)	Coefficient of variation (%)	Outliers according to Phr. Eur. 2.9.5 (%)	Outliers including damaged tablets (%)
Cylinder (d = 6 mm)	62.4 ± 3.3	4.78	0.9	8.73
Cylinder (d = 10 mm)	163.2 ± 4.5	4.94	6.7	7.78
Cylinder (d = 14 mm)	370.5 ± 3.6	4.22	19.4	19.44
Hollow cylinder	150.1 ± 4.1	4.68	5.6	6.94
Conical frustum	169.8 ± 3.9	3.93	6.5	7.94

Analyzing the 6 mm diameter cylinder, which has the highest allowable percentage deviation (10%), there is a much lower percentage of outliers among the total intact samples. The average mass of these tablets was 62.4 mg—approximately a third of the mass of the majority of the other tablets. The percentage outliers of the total intact samples were 0.9%. With such a small tablet, it was worth noting this finding, as it could be especially beneficial to be able to produce small, accurate oral dosage forms for pediatric applications. The 10 mm diameter tablets, hollow cylinder tablets, and conical frustum tablets, which all have a 7.5% weight deviation limit set for uncoated tablets in this mass category, had a similar percentage of outliers among the total intact samples. The 14 mm cylinder, which has masses exceeding 250 mg, has a weight distribution limit of 5%. These tablets had a total of 19.4% outliers of total samples, which far exceeded the percentage for any other shape analyzed in this study. While this could be due to the stricter weight distribution limit requirement, there is also a possibility that this large percentage derives from the low number of tablets printed. Due to the large size of the tablets and the limitations of the print bed size, as well as the desire to leave enough space between tablets for a good quality of print, only 12 tablets were printed per batch.

It was also observed that the tablets with mass deviations outside the allowable limit mainly had masses greater than the average mass for the 14 mm diameter cylinders. Five out of seven outliers had masses greater than the average mass. This could be caused by a combination of hot spots in the print bed and high tablet mass. These cylinders have a significantly larger size and mass than the other shapes printed, storing more thermal energy and allowing for additional sintering at the perimeter of the printed dosage forms. It could also lead to more densification of the resultant printed structure. The data indicates that large tablets are less likely to fall within the mass requirements of Phr. Eur. 2.9.5. This is not a troubling finding for the desired application, however, as it is generally recommended to produce smaller tablets for pediatric patients. While there is not a large amount of reported information detailing ingestible tablet sizes for different age groups, a former recommendation from the European Medical Agency described 15 mm and above as a very large tablet size ingestible for children between 12 and 18 years of age (Batchelor and Marriott, 2015). The 14 mm tablet is close to this diameter, and therefore not as relevant for the whole of the pediatric population.

One-way analysis of variance (ANOVA) tests were performed on the masses of the three batches for each shape created to analyze the consistency of the resultant tablets. None of the five shapes exhibited significant differences. The most consistent masses according to one-way ANOVA were exhibited by the conical frustum, 6 mm diameter cylinder, and the 14 mm diameter cylinder. These results align with the standard deviations found in Table 7, where the standard deviations for these shapes were below 4%. The standard deviation in masses observed for the other shapes created were not much higher, which aligns with the findings from one-way ANOVA suggesting no significant differences between the batches created.

From mass analysis results, it appears that the differently sized cylindrical tablets showed the largest differences when compared, though the general SA/V appears to have an impact. The outliers of the total intact samples vary greatly for the differently sized cylinders, particularly the 14 mm diameter cylinder, though the exact reason for this deviation is unknown. Additionally, the shapes with the most similarity, both in size and SA/V, are the 10 mm diameter cylinder and the conical frustum. They have a very close percentage of outliers and standard deviation on their average mass. Comparing these results to previously reported works using copovidone, several oral dosage forms printed are similar to the 10 mm diameter cylinder from this study. The reported mass in this study for the 10 mm diameter cylinder was lower than reported with use of the colorant Candurin^®^ NXT Ruby Red (Tikhomirov et al., 2023b). This is likely due to the use of a black pigment, where the laser scan speed had to be increased. It has been reported previously that lower laser scan speeds typically lead to tablets with an increased mass (Fina et al., 2018b). To further investigate the differences between the tablets, volume analysis was performed, as mass and volume are both important factors for oral dosage forms.

### 3.5 Volume analysis of tablets

To analyze the theoretical and actual volume of the different shapes and sizes of tablets, six tablets of each shape were chosen at random and the dimensions were used to determine the average actual volume of each tablet type. The theoretical volume was determined by using the intended dimensions, as previously described.

The results, seen in Table 8, were closer to that of the theoretical volumes for the cylindrical tablets than for the other shapes. This result indicates that, for this study, the more simple, cylindrical shaped tablets are more likely to conform to the intended dimensions from the STL file. The different geometries do appear to have an impact on the actual-to-theoretical volume ratio. When comparing the actual volume to the theoretical volume in other publications that use copovidone, results are mixed, with some also showing slightly higher actual volumes (Gueche et al., 2021) and others showing the actual volume slightly below the theoretical volume (Thakkar et al., 2021). This information can prove crucial for the dissolution properties and proper dosage of a tablet, making it important to discuss this aspect of the data and possibilities for why a variation between the actual-to-theoretical volume occur.

**TABLE 8:** Volume data for various tablet types.

Shape	Theoretical volume, mm^3^	Actual volume, mm^3^	Ratio of actual-to-theoretical volume
Cylinder (d = 6 mm)	113	114	1.01
Cylinder (d = 10 mm)	310	332	1.07
Cylinder (d = 14 mm)	620	672	1.08
Hollow Cylinder	200	288	1.44
Conical Frustum	240	295	1.23

The 6 mm diameter cylinder was an extremely close match, with an actual-to-theoretical volume ratio of 1.01. All other tablet types exhibited a higher actual volume than theoretical volume, though it is important to note that the ratios for the 10 mm diameter cylinder and the 14 mm diameter cylinder are closer to both each other and the 6 mm diameter tablet than the other geometries investigated in this study. While these ratios are not extremely different from the 6 mm diameter tablet, they have a slightly higher actual volume in both cases, which could be due to the larger size of these cylinders. Since these shapes are all cylinders, they should logically have the most similar proportional volumes. The hollow cylinder shows the most difference between the actual and theoretical volume. This can possibly be explained by the sintering process and smaller height of this tablet. This shape has the highest SA/V of all of the shapes in this study. Since the printer was set to sinter perimeters three times to provide a more robust tablet, the outer area of a SLS-printed tablet is generally sintered more than the interior of the tablet. For a hollow cylinder, the outer and inner border are considered perimeter by the printer and sintered multiple times. It is therefore reasonable that the actual volume would be proportionally higher reflecting the inner and outer area for this shape, as more sintering generally leads to a higher mass and volume of the tablet. Since SLS printing utilizes solid-state sintering below the melting or glass transition temperature (T_g_) of the powder materials used, the increased energy absorbed by the material from additional perimeters traced with the laser can potentially lead to a higher degree of sintering and overall energy absorbance. With more laser beam irradiation from more perimeters, there is additionally the possibility for more densification of the surrounding particles, and a resultant volume that could be greater than the theoretical, expected volume. A similar trend is seen with the conical frustum, though to a lesser extent, as the SA/V is closer to that of the cylinder. This shape also has more relative SA than a cylinder, and therefore more sintering in triplicate.

These are important aspects to consider in shape selection of tablets, as volume differences are clearly observed. These differences can potentially impact the dissolution properties of the tablets. The actual volumes observed were relatively close to that of the theoretical volumes in the case of the cylindrical shapes, indicating a consistency that is beneficial to SLS and follows previously reported literature describing SLS as having higher dimensional accuracy than other AM techniques (Kluska et al., 2018; Kozak and Zakrzewski, 2018). Therefore, further investigation is needed, with disintegration of the differently shaped and sized tablets being the next step.

### 3.6 Disintegration

Disintegration data, shown in Table 9, indicates the importance of geometry on tablets. The largest tablet in this study, the 14 mm diameter cylinder, had a significantly higher average disintegration time than the other tablets. The SA/V for this tablet was 0.79, which was the lowest of all the tablets investigated. Therefore, it is reasonable that this tablet would disintegrate more slowly. The conical frustum and the 10 mm diameter cylinder are both the most similar in dimensions and SA/V of the shapes investigated, and exhibit the most similar average disintegration time. The 6 mm diameter cylinder had a slightly higher average disintegration time than the hollow cylinder. This is interesting, as the hollow cylinder is, overall, a higher-mass, larger tablet than the 6 mm diameter cylinder. Nevertheless, it disintegrated more rapidly. The SA/V of the hollow cylinder, 1.5, is higher than the SA/V for the 6 mm diameter cylinder, 1.17, which is the probable cause of this more rapid average disintegration time. The average disintegration times and the respective SA/V for the shapes were fit to different models to see if an overall trend would emerge. The resultant best fit was an exponential decay model, which had an *R* ^2^ value of 0.96. A trend was observed from this analysis supporting that the higher the SA/V, the shorter the disintegration time.

**TABLE 9:** Disintegration results of tablets.

	Average disintegration time (s, mean ± sd)	SA/V (mm^−1^)
Cylinder (d = 6 mm)	15.3 ± 5.4	1.17
Cylinder (d = 10 mm)	19.7 ± 8.8	0.9
Cylinder (d = 14 mm)	57.5 ± 18.1	0.79
Hollow Cylinder	12.0 ± 4.5	1.5
Conical Frustum	21.7 ± 6.4	0.95

### 3.7 Dissolution of tablets

**FIGURE 8:**
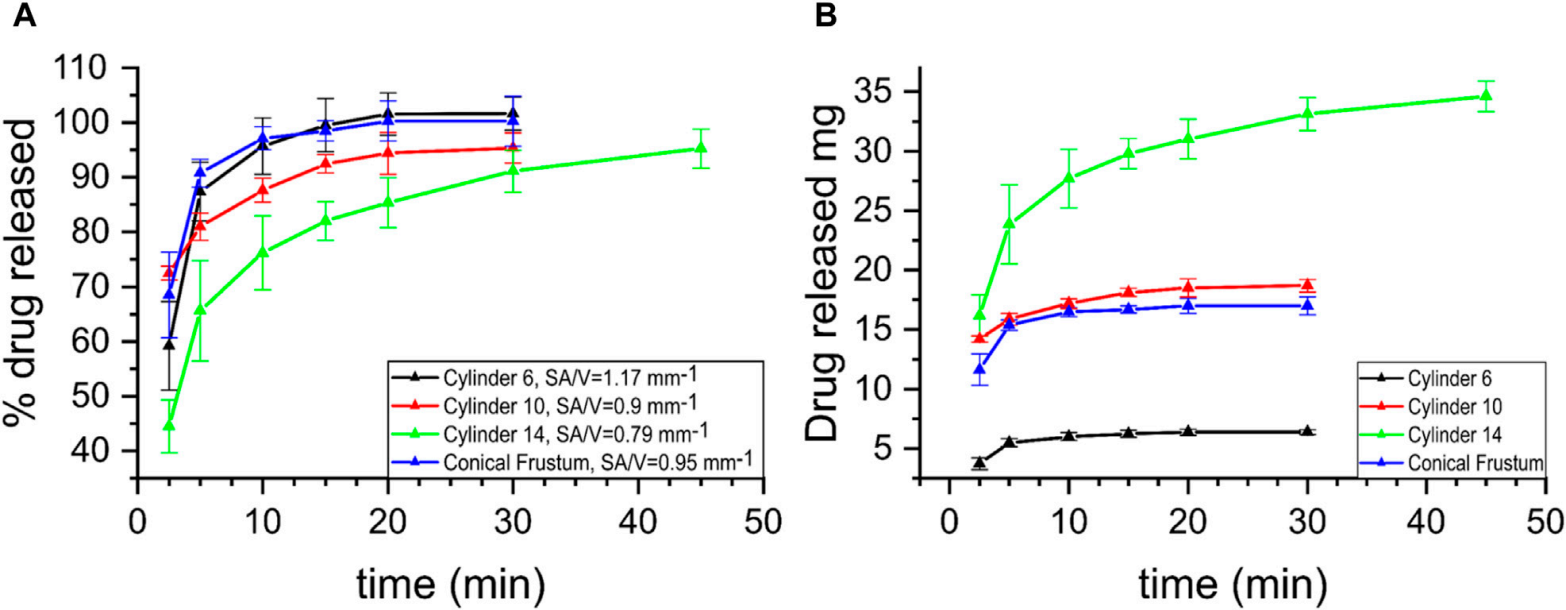
Drug release profiles of samples cylinder (d = 6 mm, SA/V = 1.17 mm^−1^; d = 10 mm, SA/V = 0.9 mm^−1^; d = 14 mm, SA/V = 0.79 mm^−1^) and conical frustum, SA/V = 0.95 mm^−1^. **(A)** % of drug release over time, **(B)** mass of drug released over time.

The release curves from the *in vitro* drug release studies are depicted in Figure 8. The different printed dosage forms contain different dosages. As described in section 2.2 Formulation Preparation, the drug content in the mixture used for printing is 10% (the same for all formulations). However, as the size and weight of the various formulations differ, so does the actual drug dosage in each of them. Nevertheless, by examining the drug release profiles of the formulations expressed both in mass of the drug released over time and percentage of the drug released over time, a more complete picture of the process can be achieved. Normalizing the drug release profiles of the formulations by expressing it as percentage of drug released over time allows for a comparison between them, even though each contains a different dose. All samples follow a similar pattern of rapid drug release, which is expected given the nature of the polymer and drug used in this study; copovidone is a fast dissolving polymer and theophylline is slightly soluble in water, c_s_ = 7.36 g/L (Yalkowsky et al., 2016) When tested for fit to kinetic models, none of the samples exhibit good fit with the Higuchi model. A possible explanation to that can be the rapidness of the drug dissolution, indicating that diffusion is not the governing factor for their release. The conical frustum and 6 mm cylinder release curves showed good fit to the first-order kinetic model with *R* ^2^ = 0.992 and 0.986 and k = 0.464 and 0.375, respectively. The curves of the cylinder samples (d = 6 mm, d = 10 mm, d = 14 mm), exhibit a slower rate of release until the plateau is reached, as the diameter is increased. This increase of diameter leads to a decrease of the SA/V, i.e. 1.17 mm^−1^, 0.9 mm^−1^ and 0.79 mm^−1^ for cylinders with diameter 6, 10, and 14 mm respectively. The latter can also explain the similar behavior between the conical frustum sample and both the cylinder d = 6 mm and d = 10 mm samples. The conical frustum sample has a drug content of ∼17 mg, whereas the cylinders d = 6 and d = 10 mm have ∼6 mg and 18 mg respectively. On the other hand, the SA/V of the conical frustum (0.95 mm^−1^) lies in-between the ones of the cylinder d = 6 mm (0.9 mm^−1^) and d = 10 mm (1.17 mm^−1^). Finally, the hollow cylinder released all of its drug content and reached 100% release within 2.5 min (data not shown). Although the drug dosage of this sample is ∼14 mg, i.e., the second lowest among all samples, its SA/V is 1.5 mm^−1^, the highest amongst them. Hence, it is suggested that a major factor governing the rate of dissolution of such systems is the aforementioned ratio. That would be in agreement with previously reported results on the effect of SA/V on the dissolution of FDM produced drug formulations (Windolf et al., 2021; Windolf et al., 2022). As noted in those works, the lower the ratio, the slower the dissolution of the drug occurs, an observation that seems to be true in SLS as well.

## 4 Conclusion

The findings from this study show that geometry clearly has an effect on characteristics of the resulting tablets. This is a factor that should be taken into consideration for the design of tablets in the future. Additionally, it is not possible to assume that the STL file will fully describe the resultant product. This study has shown that the mass and volume derived are not necessarily consistent with what is expected. While SLS is a very promising method for oral dosage form manufacturing, this study showed that different geometries can cause mass variation in each batch, as well as a trend toward slightly higher volumes than the intended STL files dictate. Some of the issues found in this study, such as print resolution and layer-to-layer adhesion, have the potential to be mitigated by dedicated pharmaceutical printers and optimized formulations. With this knowledge established, dosage form designs can be improved.

When designing tablets, it is vital to anticipate resultant changes that may occur based on shape changes. This is particularly relevant for pediatric populations, where dosages, shapes, and sizes of tablets may differ depending on a child’s age and size. Since the size of the tablets led to variations, particularly with regard to dissolution and mass uniformity, it is an important realization for children, who will need variable doses. Emphasis must be placed on the intended drug release profile, which will have a dependency on the SA and, therefore, shape of the tablet. While this aligns with findings from FDM and conventional manufacturing techniques, it is essential to have confirmed this. Assumptions between such different methods for AM cannot be made, as the AM techniques vary greatly. This study was performed with copovidone, a fast-dissolving polymer. Future studies could be performed with additional drugs and polymers, both fast and slowly dissolving, to make the results more generalizable. Further studies investigating if strongly swelling polymers behave differently than just surface eroding polymers could also be analyzed. For example, if and how pores swell shut and influence the release of disintegration. Additionally, more complex geometries, within an easy-to-swallow range, could be evaluated to find further trends that could impact tablet characteristics. With the rapid growth of SLS for pharmaceutical application and lack of in-depth investigations into the deciding factors for dosage form quality, further research such as this is mandatory. This study is a first step into the important details of geometry and the impact it has on tablets. With this knowledge, a better, safer tablet could be produced using SLS. While there is still more work to be done for SLS for pharmaceutical application to reach full maturation, an insight into the geometry effects of tablets is a step in this direction.

## Data Availability

The original contributions presented in the study are included in the article/Supplementary material, further inquiries can be directed to the corresponding authors.
